# A Single CD8^+^ T Cell Epitope Sets the Long-Term Latent Load of a Murid Herpesvirus

**DOI:** 10.1371/journal.ppat.1000177

**Published:** 2008-10-17

**Authors:** Sofia Marques, Marta Alenquer, Philip G. Stevenson, J. Pedro Simas

**Affiliations:** 1 Instituto de Microbiologia e Instituto de Medicina Molecular, Faculdade de Medicina, Universidade de Lisboa, Lisboa, Portugal; 2 Instituto Gulbenkian de Ciência, Oeiras, Portugal; 3 Division of Virology, Department of Pathology, University of Cambridge, Cambridge, United Kingdom; Oregon Health Sciences University, United States of America

## Abstract

The pathogenesis of persistent viral infections depends critically on long-term viral loads. Yet what determines these loads is largely unknown. Here, we show that a single CD8^+^ T cell epitope sets the long-term latent load of a lymphotropic gamma-herpesvirus, Murid herpesvirus-4 (MuHV-4). The MuHV-4 *M2* latency gene contains an H2-K^d^ -restricted T cell epitope, and wild-type but not M2^−^ MuHV-4 was limited to very low level persistence in H2^d^ mice. Mutating the epitope anchor residues increased viral loads and re-introducing the epitope reduced them again. Like the Kaposi's sarcoma–associated herpesvirus K1, M2 shows a high frequency of non-synonymous mutations, suggesting that it has been selected for epitope loss. *In vivo* competition experiments demonstrated directly that epitope presentation has a major impact on viral fitness. Thus, host MHC class I and viral epitope expression interact to set the long-term virus load.

## Author Summary

Persistent viruses present a major challenge to the immune response. Gamma-herpesviruses are a prime example, and the archetypal family member, Epstein-Barr virus (EBV), has been studied for many years. A major unanswered question with EBV is why long-term virus loads—a key pathogenesis outcome—vary so widely between individuals. As most EBV studies are necessarily descriptive, the murid gamma-herpesvirus MuHV-4 provides an important focus of pathogenesis research. Here, we used MuHV-4 to address what determines long-term gamma-herpesvirus loads. We find a major role for a single MHC class I–restricted latency epitope. This reflects that latency-associated viral immune evasion and transcriptional silencing create a unique setting, in which the pool of possible epitopes is small enough for epitope loss to have a significant impact on viral fitness. Our data suggest that polymorphisms in viral latency genes and in host HLA class I together determine long-term viral loads.

## Introduction

Gamma-herpesviruses characteristically persist in lymphocytes. Since the pool of latent genomes is constantly drained by viral reactivation, it must be replenished by virus-driven lymphoproliferation; this in turn is limited by host T cells; the steady-state viral load reflects an equilibrium of these fluxes. Viral loads are remarkably constant in one individual, yet vary hugely between them [1]. What determines the set point? Such questions are difficult to address without animal models. Murid herpesvirus-4 (MuHV-4) is one of the best established. It is genetically closer to Kaposi's sarcoma associated herpesvirus (KSHV) than to Epstein-Barr virus (EBV), but shares with EBV a lymphoproliferative infectious mononucleosis syndrome [2] and persistence in memory B cells [3]–[5]. The pathogenesis of KSHV infection is presumably similar. The steady state MuHV-4 latent load does not appear to reflect the inoculating virus dose [6], suggesting that it is set instead by host and viral genetic polymorphisms.

Both host immunity and viral evasion contribute to MuHV-4 pathogenesis. Evasion dominates during acute latency amplification. The MuHV-4 K3 protein promotes this [7] by degrading MHC class I heavy chains and TAP [8],[9]. K3 is transcribed in latently infected germinal centre B cells [7], but also functions in lytically infected myeloid cells [10],[11], which are present in lymphoid tissue [4] and are probably an important source of the viral M3 chemokine binding protein [12],[13]. The quantitative contribution of M3 to CD8^+^ T cell evasion remains controversial [14],[15]. However, it is clearly capable of such a role [16]. M4 is another secreted lytic gene product that promotes latency amplification [17],[18]. Thus, K3 may act both directly and by allowing lytically infected cells to protect latently infected cells in trans [19]. The MuHV-4 ORF73 episome maintenance protein has a further cis-acting CD8^+^ T cell evasion mechanism, equivalent to that of EBV EBNA-1 [20],[21], that is again vital for host colonization [22].

Despite immune evasion, virus-driven lymphoproliferation is brought under control by 3–4 weeks post-infection, at least in part by CD8^+^ T cells [23]–[25]. H2^d^ mice mount a CD8^+^ T cell response against the M2 latency gene product at this time [26]. EBV latent loads and associated pathologies are similarly controlled by CD8^+^ T cells that recognize viral latent antigens [27],[28]. EBNA-1-specific CD4^+^ T cells can also suppress EBV lymphoproliferation *in vitro* [29],[30], but whether equivalent recognition occurs *in vivo* is unclear [31]: even optimized latent CD4^+^ T cell epitope expression has little effect on MuHV-4 host colonization [32]. Most evidence would therefore suggest that gamma-herpesvirus latency is controlled principally by latent antigen-specific CD8^+^ T cells [27],[28]. Vaccination with the M2 latency epitope has little effect on MuHV-4 latency establishment [33] because viral evasion dominates this setting. However, the impact of M2 recognition on the steady state viral load has not been defined. The balance of immunity and evasion could be subtly different here, for example if M3 function is now blocked by antibody.

M2 itself promotes acute latency amplification [34]–[36] by modulating Vav-dependent B cell signaling [37],[38]. The EBV LMP-2A [39],[40] and KSHV K1 [41],[42] have equivalent roles. M2 also has anti-interferon and anti-apoptotic functions [43],[44], although what these contribute to latency is unclear. An unusual feature of the M2 knockout phenotype in BALB/c (H2^d^) mice is that despite an acute latency deficit, long-term latency is increased [36]. C57BL/6 mice (H2^b^), which are not known to recognize an M2 epitope, show the same acute latency deficit, but not the long-term increase [34]. Here we show that although M2 itself promotes acute latency establishment, its H2-K^d^-restricted CD8^+^ T cell epitope is a major negative determinant of the long-term viral load. The reduction in latency associated with M2 expression required both its CD8^+^ T cell epitope and an appropriate host restriction element. Thus, host MHC class I polymorphisms interact with viral latency gene expression to determine the steady state gamma-herpesvirus load.

## Results

### Long-term latency depends on viral M2 expression and host H2 haplotype

**Figure 1:**
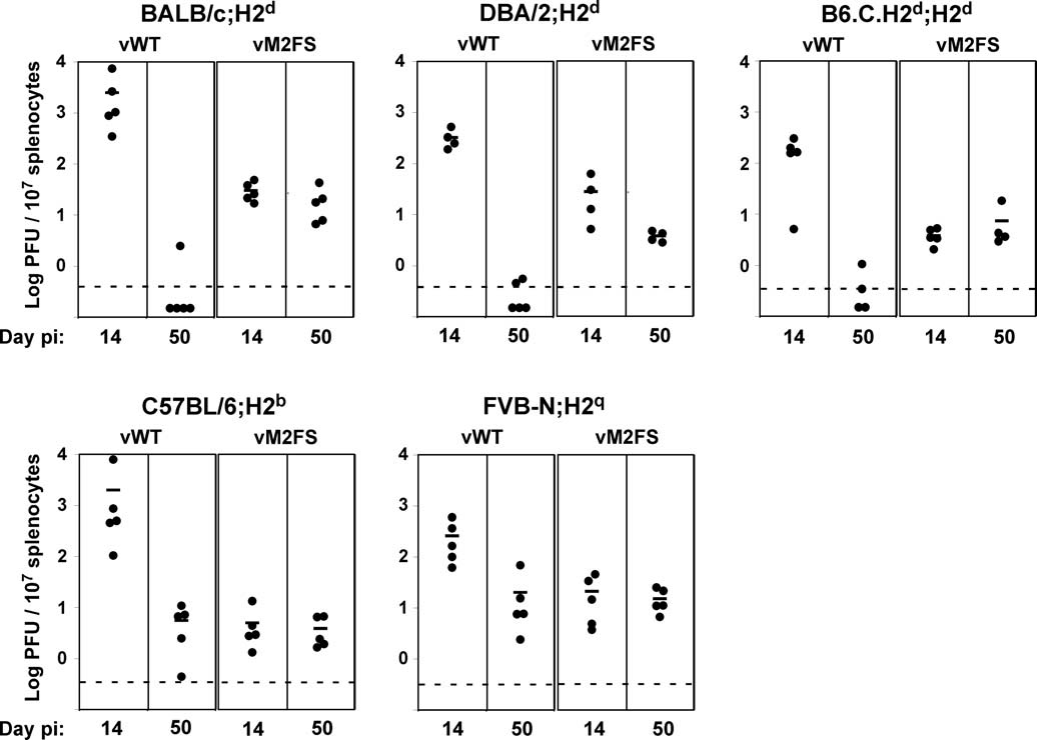
Long-term MuHV-4 latent loads depend on viral M2 expression and host H2 haplotype. Mice were infected intranasally with wild-type (vWT) or M2^−^ (vM2FS) viruses. At d14 or d50 post-infection spleens were removed and titrated for reactivation-competent virus by explant co-culture with BHK-21 cells. Each point shows the titre of one mouse. The horizontal bars show arithmetic means. The dashed horizontal line represents the limit of assay detection. Pre-formed infectious virus, as measured by the parallel titration of equivalent frozen/thawed samples, was below the limit of assay detection. At d50 post-infection the vM2FS latent load was significantly higher than the vWT latent load in BALB/c (P = 0.011) and DBA/2 (P = 3×10^−5^) and close to significance in B6.C.H2^d^ (P = 0.056), but not in C57BL/6 (P = 0.228) and FVB-N (P = 0.347) by a one-tailed Student's t-Test. This was also confirmed by combining all data from H2^d^ mouse strains (P<0.0001) but not from non-H2^d^ mice (P = 0.71) using a 2-way non-parametric ANOVA Friedman's test. Data were reproducible over two independent experimental groups.

Our starting point were the observations that MuHV-4 M2 knockouts show an acute latency deficit in both BALB/c and C57BL/6 mice, but an elevated long-term latent load only in BALB/c mice [34],[36]; and that mutating only the M2 amino acid residues critical for its interactions with Vav and Fyn [37],[38] reproduces the acute latency deficit but not the long-term increase [37]. We hypothesized that a lack of H-2K^d^-restricted epitope presentation might contribute significantly to the M2 knockout phenotype. We tested this further by comparing M2^−^ (vM2FS; a previously described M2 frame shift mutant [36]) and M2^+^ (vWT) viral loads in different H2^d^ and non-H2^d^ mice (Figure 1).

At 14 days post-infection, both H2^d^ and non-H2^d^ mice showed an M2-dependent latency deficit, consistent with M2 having an important role in acute latency amplification, when viral evasion limits CD8^+^ T cell function [7],[14],[22]. But by 50 days post-infection, when reactivatable wild-type virus was barely detectable in BALB/c mice (H2^d^), M2^−^ virus titres were maintained and now exceeded those of the wild-type. In contrast, long-term vWT titres in C57BL/6 mice (H2^b^) were equivalent to those of the M2 mutant; DBA/2 mice (H2^d^) were similar to BALB/c; FVB-N (H2^q^) were similar to C57BL/6; and B6.C mice, where the H2^d^ locus has been backcrossed onto a C57BL/6 background, were similar to BALB/c. M2 expression therefore increased the acute latent load independent of H2 type and reduced the long-term latent load in an H2-restricted manner: low long-term latency levels correlated with the H2^d^ haplotype.

### Generation of M2 mutant viruses with altered H-2K^d^ epitopes

**Figure 2:**
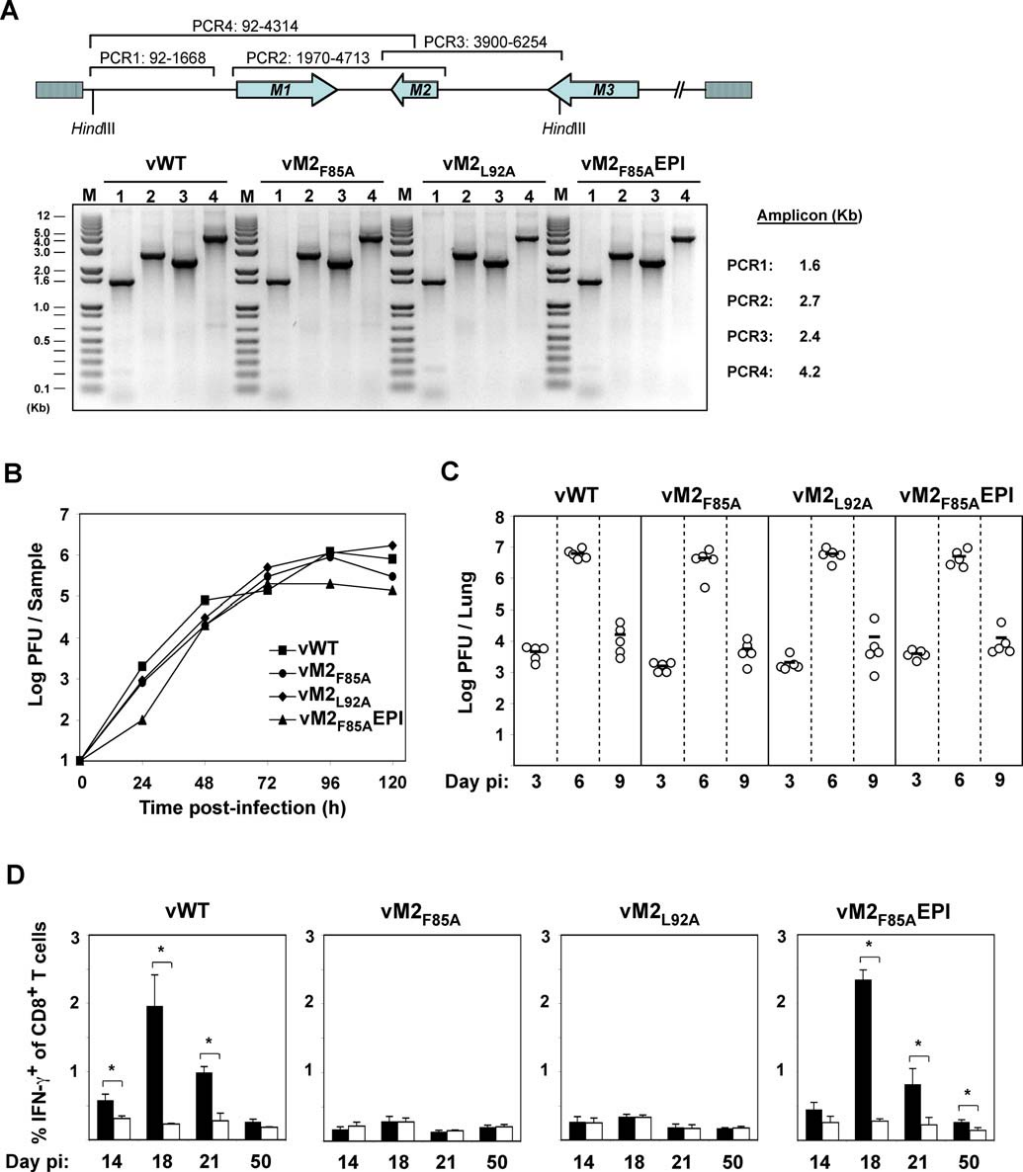
MuHV-4 latent epitope mutants show normal in vitro and in vivo replication. A. High molecular weight DNA from MuHV-4-infected BHK-21 cells was checked by PCR for genome integrity in the HinDIII-E region. A schematic representation of the MuHV-4 genome and amplicon coordinates for each PCR is shown. B. BHK-21 cells were infected (0.01 PFU per cell) with the indicated viruses, washed in PBS and virus replication with time was monitored by plaque assay. C. BALB/c mice were intranasally infected with 10^4^ PFU of the indicated viruses. At the indicated days post-infection lungs were removed and titrated for infectious virus by plaque assay. Each point represents the titre of an individual mouse. Horizontal lines indicate arithmetic means. None of the mutants showed a deficit relative to the wild-type (p>0.5 by 1-way non-parametric ANOVA Kruskal-Wallis Test). D. Splenocytes of BALB/c mice infected with viruses of the indicated genotypes were stimulated in vitro with either M2_84–92_ (black bars) or EGFP_200–208_ as a control (white bars) in the presence of Brefeldin A, then stained for intracellular interferon-gamma. The data show the percentage of CD8^+^ T cells responding to peptide at each time point (arithmetic means±SEMs from 3 independent measurements). *, p<0.05 using a 1 tailed Student's t-test.

Residues 84–92 of M2 contain its H-2K^d^-restricted T cell epitope, GFNKLRSTL [26]. We tested whether the recognition of this epitope could explain the H2-restricted difference in long-term M2^−^/M2^+^ latent loads by mutating its anchor residues to alanines, either the phenylalanine at position 85 (vM2_F85A_) or the leucine at position 92 (vM2_L92A_). We reverted the vM2_F85A_ mutant in two ways: first conventionally, by restoring position 85 to phenylalanine (vM2_F85A_R), and second by re-introducing the GFNKLRSTL epitope ectopically at the M2 C-terminus (vM2_F85A_EPI). All these viruses showed an otherwise intact M2 locus, normal *in vitro* growth and normal replication in infected lungs (Figure 2A–C). Intracellular IFN-γ staining of CD8^+^ T cells from infected mice (Figure 2D) showed that either anchor residue mutation prevented the generation of H-2K^d^-GFNKLRSTL-specific CD8^+^ T cells, and that re-introducing the epitope into its ectopic site restored the response.

### Long-term latent loads of M2_84–92_ anchor residue mutants

**Figure 3:**
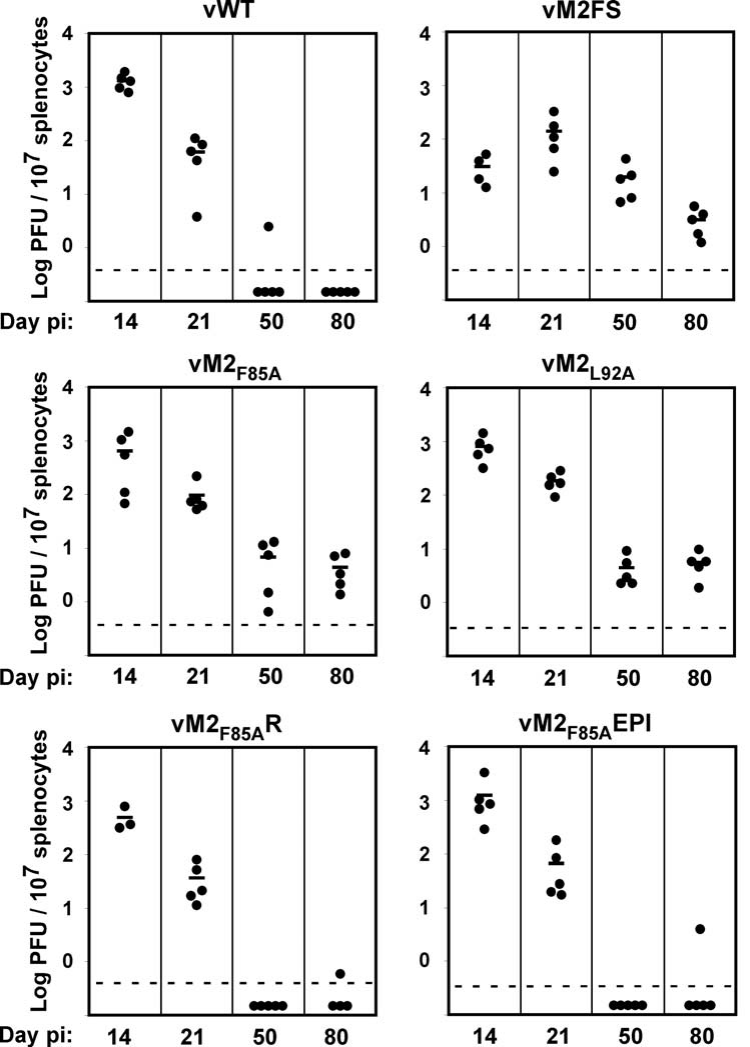
The presence of a CD8^+^ T cell epitope in M2 sets the MuHV-4 long-term latent load. BALB/c mice were intranasally infected (10^4^ PFU) and at 14, 21, 50 or 80 days post-infection spleen removed and titrated for reactivation-competent virus by explant co-culture. Each point shows the titre of one mouse. Horizontal lines indicate arithmetic means. The dashed horizontal line represents the limit of assay detection. Pre-formed infectious virus, as measured by parallel titration of equivalent frozen/thawed samples, was below the limit of detection of the assay. At d50 and d80 post-infection vM2_F85A_ and vM2_L92A_ showed significantly higher latency loads than vWT: d50, vM2_F85A_ p = 0.021, vM2_L92A_ p = 0.012; d80, vM2_F85A_ p = 0.007, vM2_L92A_ p = 0.001; using 1 tailed Student's t-Test with p values shown when false discovery rate correction for multiple testing (FDR) was <0.05. Data were reproducible over two independent experimental groups.

The acute (d14) latency titres of the anchor residue mutants in BALB/c mice were indistinguishable from the wild-type (Figure 3). Thus, there was no evidence that the point mutations affected M2 function. This was consistent with neither residue 85 nor residue 92 being crucial for the M2 Vav/Fyn interaction [37],[38]. The C-terminal GFNKLRSTL epitope insertion also had no appreciable impact on latency establishment. At d14 post-infection, the impact of epitope presentation is limited by viral evasion. But in contrast to these normal acute titres, the long-term titres of the anchor residue mutants were increased, like those of the vM2FS mutant, while those of the vM2_F85A_R, vM2_F85A_EPI viruses were low, like those of the wild-type. Thus, the presence of a presentable epitope in M2 had a major impact on the long-term viral load.

### Lower latency loads due to M2_84–92_ epitope expression are CD8^+^ T cell linked

**Figure 4:**
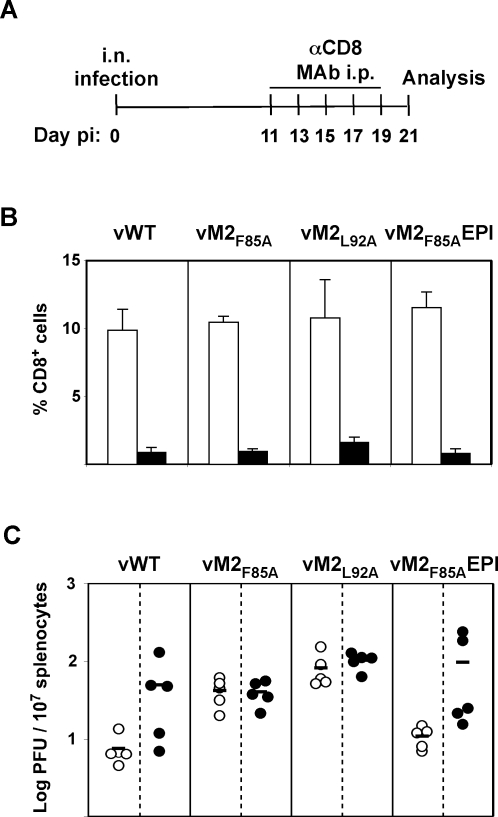
M2_84–92_ epitope expression and CD8^+^ T cells are linked in setting splenic latent loads. BALB/c mice were intranasally infected with the indicated viruses (10^4^ PFU) and at 11, 13, 15, 17 and 19 days post-infection anti-CD8 monoclonal antibody (MAb) was intraperitoneally injected. At 21 days post-infection spleens were removed for analysis. Control mice are d21 infected littermates that were not injected with MAb. A. Schematic diagram of the experimental setting. B. Splenocytes were stained for CD8. The data show the percentage of CD8^+^ T cells of total lymphocytes (arithmetic means±STDV) in depleted mice (black bars) and control mice (white bars). C. Splenocytes were titrated for reactivation-competent virus by explant co-culture. Each point shows the titre of one mouse. Black symbols represent data for CD8^+^ T cell depleted mice, white symbols represent data for control mice. Horizontal lines indicate arithmetic means. Data were reproducible over two independent experimental groups.

The increase in long-term viral load following CD8^+^ T cell epitope disruption implied that CD8^+^ T cell function helps to set this load in BALB/c mice. To confirm this, we depleted CD8^+^ T cells from vWT infected BALB/c mice by injection of anti-CD8 monoclonal antibody (MAb). Importantly, depletion was initiated at 11 days post-infection, which is after the resolution of lytic infection but prior to the peak H-2K^d^-GFNKLRSTL-specific CD8^+^ T cell response. The last MAb injection was performed at d19. Latent loads were analysed at d21 post-infection (Figure 4). The variability in titer between depleted mice probably reflected incomplete depletion, as post-infection depletions are often less efficient than pre-infection (our unpublished data), and the efficacy of depletion in individual mice infected with vWT or vM2_F85A_EPI correlated with viral load. Nevertheless, mice infected with the anchor residue mutants had significantly higher splenic latent loads than mice infected with epitope-expressing viruses before depletion, and not after CD8^+^ T cell depletion. CD8^+^ T cells were therefore responsible for the low latent loads of vWT and vM2_F85A_EPI.

### Increased germinal centre B cell colonization by MuHV-4 lacking M2_84–92_ epitope anchor residues

**Figure 5:**
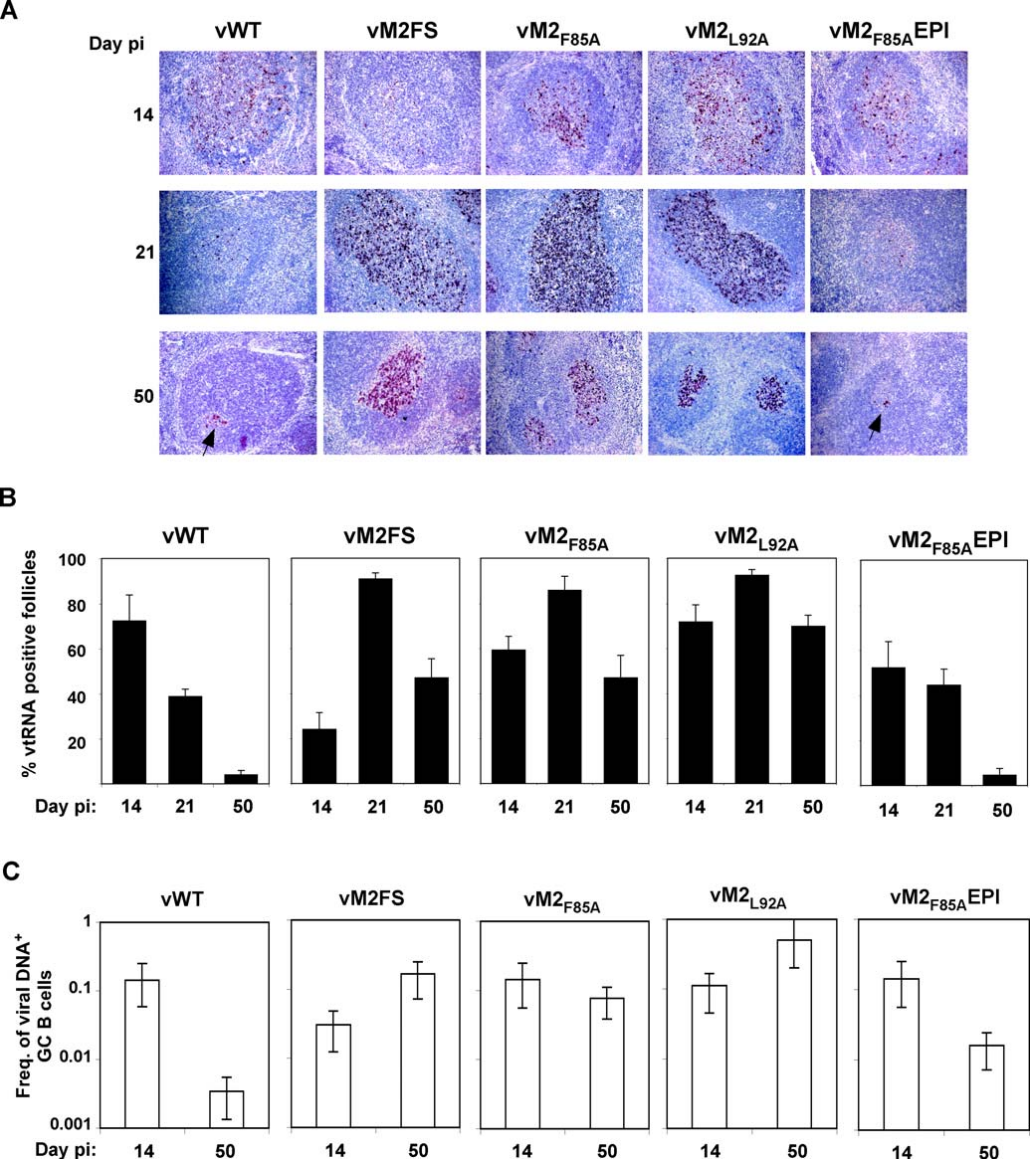
Increased germinal center B cell colonization by MuHV-4 lacking M2_84–92_ epitope anchor residues. BALB/c mice were intranasally infected as shown and spleen sections hybridized with viral tRNA-specific riboprobes. A. Representative spleen sections from each group of animals. Dark staining cells express viral tRNAs. Arrows indicate example positive cells. All sections are magnified ×200 and counter stained with haematoxylin. B. Mean±SEM percentage of splenic follicles positive for viral tRNA expression. Six sections per mouse and three mice per group were counted at each time point. Follicles were scored positive if they contained at least one viral tRNA positive cell. At d50 post-infection vM2_F85A_ and vM2_L92A_ showed significantly higher latency loads than vWT (p = 0.002 and p<0.001; using a 1 tailed Student's t-Test), whereas vM2_F85A_EPI showed no significant difference (p = 0.45). C. BALB/c mice were intranasally infected with 10^4^ PFU of the indicated virus. At 14 or 50 days post-infection, reciprocal frequencies of viral infection in flow cytometrically purified germinal center (B220^+^PNA^high^) B cells were determined by limiting dilution and real time PCR. Spleens were pooled from 5 mice per group. Bars show the frequency of viral DNA positive cells with 95% confidence intervals.

*In situ* hybridization for viral tRNA expression, a marker of lymphoid colonization [45],[46] (Figure 5A–B), showed similar results to the explant co-culture assays. Thus, the wild-type signal was high acutely (d14) but low long-term; the vM2FS mutant had a low acute signal but was higher at later times; the anchor residue mutants had high signals both acutely and long-term; and re-introducing the GFNKLRSTL epitope reduced the long-term signal back to wild-type levels. The vM2_F85A_ and vM2_L92A_ mutants had both more viral tRNA^+^ germinal centres (Figure 5B) and bigger viral tRNA^+^ germinal centres (Figure 5A), consistent with the idea that disrupting CD8^+^ T cell recognition of M2 allowed more extensive proliferation of latently infected B cells.

We have previously correlated the higher long-term latent loads of M2^−^ MuHV-4 in BALB/c mice with increased frequencies of viral genome^+^ germinal centre B cells [36]. We tested whether this applied also to the anchor residue mutants by subjecting flow cytometrically sorted germinal centre B cells to limiting dilution, PCR-based viral genome detection (Figure 5C). The frequency of viral genome^+^ B cells was higher for the wild-type than for the vM2FS mutant at 14 days post-infection, and lower at 50 days post-infection; the vM2_F85A_ and vM2_L92A_ mutants showed high frequencies of viral genome^+^ B cells at both time points; and the vM2_F85A_R (data not shown) and vM2_F85A_EPI revertants were similar to the wild-type. Moreover, even at 133 days post-infection 1 in 19 GC B cells carried M2_F85A_ DNA. At this time the frequency of M2_F85A_R DNA^+^ GC B cells was 1 in 5274. Thus, the colonization of germinal centre B cells matched the total viral load in the spleen, with early colonization depending on M2 function and late colonization depending on T cell epitope presentation.

### Evidence for M2 positive selection

M2 is positionally homologous to the KSHV K1. In so far as both modulate B cell antigen receptor signalling [37],[38],[41],[42], they are also functionally homologous. Thus, it might be expected that MuHV-4 and KSHV share a latency program where M2 or K1 accounts for much of the presentable latent antigen. There is indirect evidence that this matters for K1: DNA sequence comparison between KSHV strains suggests that K1 has been positively selected for amino acid diversity [47]. A comparison of MuHV-4 with a closely related herpesvirus recovered from a shrew [48] shows the same phenomenon: M1, M3, M4 and ORF4 have non-synonymous to synonymous mutation ratios of 0.20–0.27, while M2 has a ratio of 1.01 (Andrew Davison, personal communication).

**Figure 6:**
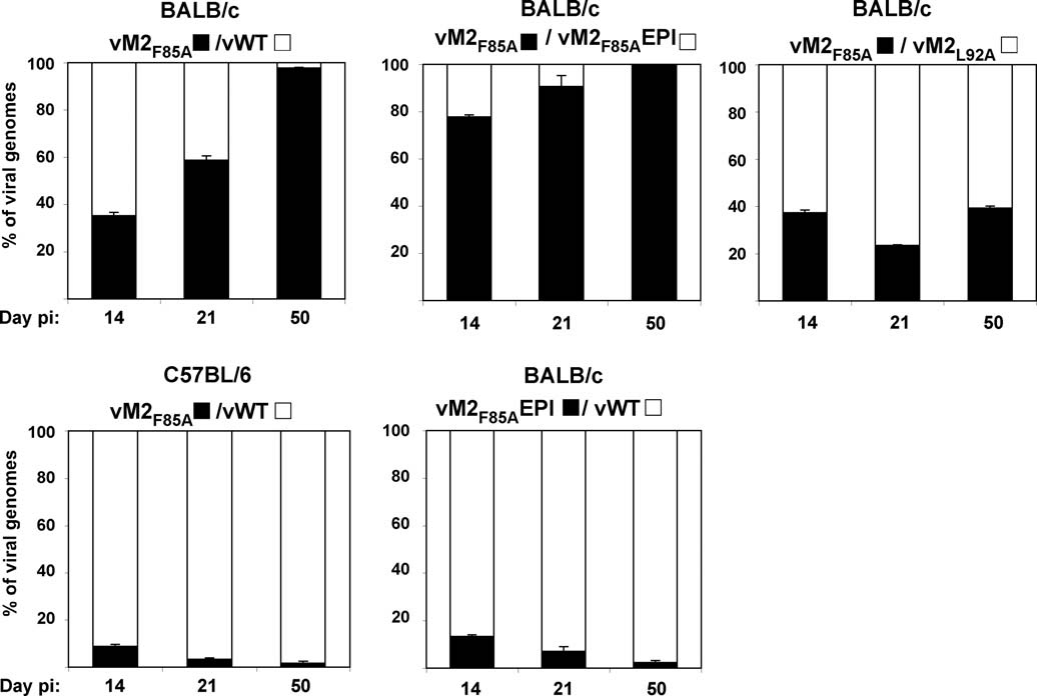
MuHV-4 lacking the H2-K^d^-restricted M2 CD8^+^ T cell epitope is positively selected in a H2^d^ host. BALB/c or C57BL/6 mice were intranasally infected with equal amounts of the indicated viruses, 10^4^ PFU in total. At 14, 21 or 50 days post-infection germinal centre (B220^+^PNA^high^) B cells were recovered from pools of al least three spleens by flow cytometric sorting. The copy number of each viral genome was then measured by quantitative PCR using primers specific for each viral genome. Each sample was assayed in triplicate. Black bars denote the percentage of M2_F85A_ or vM2_F85A_EPI genomes in the total genome load. Error bars show 95% confidence intervals.

In order to gain more direct evidence for M2 immune selection, we co-infected BALB/c mice with epitope^+^ and epitope^−^ viruses, and quantified by real-time PCR viral genome loads in germinal centre B cells using virus-specific primers (Figure 6). For co-infection experiments, aliquots of the same viral preparations were used to formulate viral mixes that contained equal amounts of infectious units of each virus of interest, i.e. 5×10^3^ PFU of each viral genotype, as determined by plaque assay. At d14 post-infection, there was little difference between vWT and vM2_F85A_, but by d50 vM2_F85A_ accounted for >95% of the viral genomes. vM2_F85A_EPI and vM2_F85A_ gave a similar result, the proportion of vM2_F85A_ genomes increasing with time, while mixed vM2_F85A_ and vM2_L92A_ loads remained equivalent. Thus, viruses lacking M2 epitope presentation contributed disproportionately to long-term host colonization in BALB/c mice. The advantage of epitope null viruses over vWT was H2^d^-dependent, since it was not observed in C57BL/6 mice co-infected with vWT and vM2_F85A_. Here, vWT accounted for the majority of MuHV-4 genomes at all times.

## Discussion

Gamma-herpesvirus infections encompass complex combinations of cell types, anatomical sites, viral gene expression patterns and immune effector functions. This has made elusive a comprehensive understanding of how host immunity and viral evasion interact. Nevertheless, a consensus picture is now emerging. The long-term latent viral load is a key outcome, since it correlates with virus shedding [1] and probably also with disease. Maintained episomes replicate in step with normal cell division [49]. However, compensating for reactivation-associated latent genome loss requires a more complex program of virus-driven lymphoproliferation. This opens up another front between host immunity and viral evasion.

Lytic reactivation could itself potentially re-seed latency, and this seems to be important in B cell-deficient mice [50]. However, these mice lack both the major MuHV-4 latency reservoir and virus-specific antibody, and consequently have an infection quite different to that of wild-type mice. The severe latency deficiency of viruses lacking episome maintenance [51],[52] argues that as with EBV [53], MuHV-4 persistence in immunocompetent hosts depends on lymphoproliferation. The efficiency with which proliferating B cells present CD8^+^ T cell targets must therefore also be important. The protection of B cells expressing M2 by K3 is probably only partial. The data presented here show that the impact of CD8^+^ T cell immunity can depend on a single epitope, consistent with epidemiological evidence of epitope selection in the EBV EBNA-3 [54],[55] and the KSHV K1 [47].

The importance of a single latency epitope for MuHV-4 contrasts with lymphocytic choriomeningitis virus infection, where removing an immunodominant CD8^+^ T cell target simply brings out subdominant epitopes [56]. This may reflect that lymphocytic choriomeningitis virus does not suppress MHC class I-restricted antigen presentation, making the pool of possible epitopes larger. C57BL/6 mice illustrate what can happen when classical CD8^+^ T cell recognition of a key MuHV-4 target fails. Rather than overt disease, a back-up mechanism of non-classical Vβ4^+^CD8^+^ T cell recognition comes into play [17],[57]. The higher latent loads of C57BL/6 mice despite massive CD8^+^Vβ4^+^ T cell expansion suggest this mechanism is not particularly efficient, and in C57BL/6 mice infected with K3-deficient MuHV-4 [7] or in BALB/c mice infected with the wild-type [17] CD8^+^Vβ4^+^ T cell expansion is minimal, presumably because classical recognition takes over. However, non-classical recognition appears to provide a safety net when host genetics or viral evasion limit normal antigen presentation.

Unlike the fairly consistent and predictable effects of an attack on the cellular antigen presentation machinery or a silencing of viral transcription/translation, the interaction between viral epitope loss and host MHC class I diversity creates unstable and hard-to-predict outcomes. For example, the most pathogenic virus variant may be quite different between different out-bred hosts. Epitope selection may even allow gamma-herpesviruses contracted from close relatives to establish higher average latent loads than those from MHC class I-incompatible strangers. The data presented here argue that small variations in key viral latency genes can have major impacts on pathogenesis, and must therefore be considered in any attempt to understand the infection of individual hosts.

## Materials and Methods

### Cell culture and viruses

NIH-3T3-CRE cells [7] were grown in Dulbecco's modified Eagle's medium (DMEM) supplemented with 10% fetal bovine serum, 2 mM glutamine, 100 U/ml penicillin and 100 µg/ml streptomycin. Baby hamster kidney cells (BHK-21) were cultured in Glasgow's modified Eagle's medium supplemented as above plus 10% tryptose phosphate broth. Murid gammaherpesvirus 4 (MuHV-4) strain 68 was used in this study [58]. To prepare viral stocks, low multiplicity infections (0.001 PFU per cell) of NIH-3T3-CRE or BHK-21 cells were harvested after 4 days and titred by plaque assay [59].

### Recombinant viruses

The vM2FS [36], vM2_F85A_, vM2_L92A_ and vM2_F85A_EPI viruses were derived from BAC-cloned MuHV-4 [60]. The M2_F85A_ and M2_L92A_ mutations were generated by overlapping PCR: A_4353_G, A_4354_C substituted alanine for phenylalanine at M2 position 85, and A_4332_G, A_4333_C substituted alanine for leucine at position 92. Mutated genomic fragments were inserted into a HinDIII-E genomic clone [61] cloned in pST76K-SR shuttle plasmid [60] using BlnI (nt 3908) and XhoI (nt 5362) restriction sites. To generate vM2_F85A_EPI, a genomic HinDIII/XhoI fragment (nt 4029–5362) was PCR-amplified from an M2_F85A_ template and cloned into pSP72 (Promega). Genomic co-ordinates 3846–4029 were then amplified, using the primer 5′-AAAAAGCTTAGGGGATTCAATAAACTTAAGTCGACGTTATAACAGTGAAGGTGCTAACGCAGAA-3′ and cloned into the same vector as a BglII/HindIII fragment, thereby attaching the amino acid residues KLRGFNKLRSTL to the M2_F85A_ C-terminus. This construct was again subcloned into the HinDIII-E shuttle plasmid using BlnI/XhoI restriction sites. A vM2_F85A_ revertant virus (vM2_F85A_R) was generated using a wild-type HinDIII-E genomic clone. All PCR-derived regions were sequenced to confirm the integrity of the mutations. Each HinDIII-E shuttle plasmid was transformed into DH10B *E.coli* containing the wild type MuHV-4 BAC. Following recombination, mutated BAC clones were identified by DNA sequencing. The integrity of each BAC was confirmed by restriction digestion with BamHI and EcoRI. All viruses were reconstituted by transfecting BAC DNA into BHK-21 cells using FuGENE 6 (Roche Molecular Biochemicals). The *loxP*-flanked BAC cassette was then removed by viral passage through NIH-3T3-CRE cells. The integrity of each reconstituted virus was checked by PCR of viral DNA across the HinDIII-E region. The stability of the introduced mutations was confirmed by viral DNA sequencing across *M2*, both prior to infection and using viruses recovered from infected mice.

### In vivo infections and virus assays

6- to 8-week old BALB/c, C57BL/6, DBA/2, FVB-N (Instituto Gulbenkian de Ciência, Portugal) and B6.C.H2^d^ mice (kindly provided by C. Penha-Gonçalves, Instituto Gulbenkian de Ciência, Portugal) were inoculated intranasally with 10^4^ PFU of MuHV-4 under isofluorane anaesthesia. At different days post-infection, lungs or spleens were removed for post-mortem analysis. Titres of infectious virus were determined by plaque assay of freeze-thawed tissue homogenates on BHK-21 cells. Latent virus loads were quantified by explant co-culture of freshly isolated splenocytes with BHK-21 cells. Plates were incubated for 4 (plaque assays) or 5 (explant co-culture assays) days, then fixed with 4% formal saline and counterstained with toluidine blue for plaque counting.

### CD8^+^ T cell depletions

MuHV-4 infected BALB/c mice were depleted of CD8^+^ T cells by 5 intraperitoneal injections of 200 µg of monoclonal antibody YTS 169.4 [62]. Blood samples or splenocytes from depleted or control mice were stained with APC-conjugated anti-CD8α and phycoerythrin-conjugated anti-CD4 (BD Pharmingen) and analysed on a FCAScan Flow Cytometer using CellQuest software (Becton Dickinson Immunocytometry systems).

### In vitro T cell stimulation

Spleen cells (1–2×10^6^) were stimulated (6 h, 37°C) with 1 µM GFNKLRSTL (M2_84–92_) or HYLSTQSAL (EGFP_200–208_) peptides (SIGMA-Genosys, Haverhill, UK) in RPMI supplemented with 10% fetal bovine serum, 2 mM glutamine, 100 U/ml penicillin, 100 µg/ml streptomycin, 50 µM 2-mercaptoethanol, 10 U/ml recombinant murine IL-2 (PeproTech, UK) and 10 µg/ml Brefeldin A. The cells were then washed in PBS/10 µg/ml Brefeldin A, blocked with anti-CD16/32 mAb, stained with APC-conjugated anti-CD8a (BD Pharmingen), washed twice, fixed in 2% paraformaldehyde (30 min, 4°C), washed once, permeabilized with 0.5% saponin, washed once, stained with a phycoerythrin-conjugated anti-interferon-gamma mAb (BD Pharmingen) and washed twice. All cells were analysed on a BD FACSCanto Flow Cytometer using FACSDiva software (BD Biosciences).

### Limiting Dilution analysis

The frequency of MuHV-4 genome-positive germinal centre B cells was determined by limiting dilution and real-time PCR [37]: B220^+^PNA^high^ B cells were recovered from pools of five spleens using a BD FACSAria Flow Cytometer (BD Biosciences) and serially two fold diluted. Eight replicates of each dilution were analysed by real time PCR (ABI Prism 7000 Sequence Detection System, Applied Biosystems). The primer/probe sets were specific for the MuHV-4 ORF65 gene (5′ primer: GCCACGGTGGCCCTCTA; 3′ primer: CAGGCCTCCCTCCCTTTG; probe: *6-FAM*-CTTCTGTTGATCTTCC–*MGB* ). Samples were subjected to a melting step of 95°C for 10 min followed by 40 cycles of 15 s at 95°C and 1 min at 60°C. Real-time PCR data was analysed on the ABI Prism 7000 software. The purity of sorted cells was always greater than 97.5%.

### *In situ* hybridization

*In situ* hybridization with a digoxigenin-labelled riboprobe encompassing MuHV-4 vtRNAs 1–4 and microRNAs 1–6 was performed on formalin-fixed, paraffin-embedded spleen sections [46]. Probes were generated by T7 transcription of a pEH1.4 (Roche Molecular Biochemicals). Positive follicles were scored using a Leica DM 5000B microscope.

### Viral genome quantification

Splenic germinal centre B cells (B220^+^PNA^high^) were obtained from pools of three spleens using a BD FACSAria Flow Cytometer (BD Biosciences) and lysed overnight in 0.45% Tween-20, 0.45% NP-40, 2 mM MgCl_2_, 50 mM KCl, 10 mM Tris-HCl pH = 8.3 and 0.5 mg/ml Proteinase K. Individual viral genomes (vM2_F85A_/vWT; vM2_F85A_/vM2_L92A_; vM2_F85A_/vM2_F85A_EPI or vM2_F85A_EPI/vWT) were quantified by real time PCR (RotorGene 6000 5-plex HRM, Corbett Research), using a labeled probe specific for *M2* and a common primer plus mutant-specific primer. vM2_F85A_/vWT viral mix: probe- 6-FAM-CATGGGGACTTTAACGTCGACCTAAGTT-TMR; common primer-GGTTAACTTCTTCAGGACTTGGTACA; M2_F85A_ specific primer-TCCTAAAACCATAAGAAGGGGAGC; WT specific primer-TTTCCTAAAACCATAAGAAGGGGATT; vM2_F85A_/vM2_L92A_ viral mix: probe- 6-FAM-TCCCCTTCTTATGGTTTTAGGAAAGCGA-TMR; common primer-CATCCCTCAGGAAATAAAAACAGTTC; M2_F85A_ specific primer-GGCTTCCATGGGGACTTTAA; M2_L92A_ specific primer-GCTTCCATGGGGACTTTGC; vM2_F85A_/vM2_F85A_EPI or vM2_F85A_EPI/vWT viral mixes: probe- 6-FAM-CCCCATGAACCCTGAGATACGTCTTCCT-TMR; common primer-TGGCTCGACTGACAGTCCAGA; M2_F85A_ and WT specific primer ACCTAAGTTTATTGAATCCCCTAAGC; M2_F85A_EPI specific primer-GTCGACCTAAGTTTATTGAATCCCCT (all primers and probes from TIBMolbiol). Samples were subjected to a melting step of 95°C, 5 min followed by 45 cycles of 15 s at 95°C and 45 s at 65°C. The wild-type, M2_F85A_, M2_L92A_ or M2_F85A_EPI HinDIII-E shuttle plasmids were used as templates to derive standard curves. Real-time PCR data was analysed using Rotor-Gene 6000 Series Software.

### Statistical analysis

Data comparisons between different infection groups were performed using Student's t-Test, Friedman's Test or the Kruskal-Wallis Test as appropriate. For limiting dilution analysis 95% confidence intervals were determined as previously described [36].
